# Public health round-up

**DOI:** 10.2471/BLT.23.010823

**Published:** 2023-08-01

**Violence against women and girls in Sudan**

Children at an internally displaced persons facility in Madani, Al Jazira State, Sudan. Thousands of people have sought shelter from the fighting that broke out in the country on 15 April and assaults, including sexual assaults, on internally displaced and refugee women and girls have risen sharply. Senior officials at United Nations agencies called for an immediate end to the violence on 5 July, and urged parties to the conflict to meet their obligations to protect civilians as per international humanitarian and human rights laws.

**Figure:**
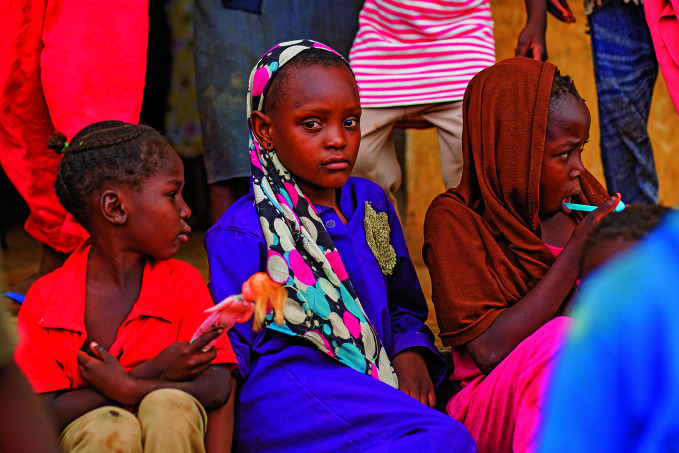
UNICEF/Ahmed Elfatih Mohamdeen

## Avian influenza concerns

Recent changes in the ecology and epidemiology of avian influenza, including a sharp rise in mammalian cases, are heightening concerns regarding the disease’s potential risk to humans.

According to a situation analysis released on 12 July by the World Health Organization (WHO), the Food and Agriculture Organization (FAO) and the World Organisation for Animal Health (WOAH), ongoing outbreaks of avian influenza are devastating animal populations, including populations of poultry, wild birds, and some mammals.

The increasing number of H5N1 avian influenza cases among the mammals is of particular concern because of the increased likelihood of human infection, and the possibility of adaptations occurring in the mammalian population leading to the emergence of new viruses that could be more harmful to animals and humans.

“With the information available so far, the virus does not appear to be able to transmit from one person to another easily, but vigilance is needed to identify any evolution in the virus that can change that,” said Dr Sylvie Briand, Director of Epidemic and Pandemic Preparedness and Prevention at WHO.

WHO is working closely with the FAO, WOAH and laboratory networks to monitor the evolution of the viruses, looking for signals of any change that could be more dangerous to humans.

https://bit.ly/3DdL3Du

## Gender-based violence in Sudan

Senior officials at United Nations (UN) agencies called for an immediate end to gender-based violence in Sudan.

In a joint statemen issued on 5 July, the heads of the UN Office for the Coordination of Humanitarian Affairs, the UN Human Rights Office, the UN Refugee Agency, the UN Children’s Fund, the UN Population Fund, UN Women and WHO drew attention to increased reports of gender-based violence, including conflict-related sexual violence against internally displaced and refugee women and girls, and called for thorough, impartial investigations and for perpetrators to be held accountable.

The agency heads stressed that all parties to the conflict must meet their obligations under international humanitarian law and human rights law to protect civilians, including women and girls, including allowing safe passage for survivors to access health care and for health workers to reach health facilities.

Even before fighting broke out on 15 April, more than 3 million women and girls in Sudan were at risk of gender-based violence, including intimate-partner violence, according to UN estimates. This number has since climbed to an estimated 4.2 million people.

https://bit.ly/43hL6sw

## Action on climate and health

Countries of the WHO European Region made commitments to addressing wide-ranging health challenges related to climate change, environmental pollution, biodiversity loss and land degradation.

Gathering at the 7th Ministerial Conference on Environment and Health in Hungary, on 7 July the countries adopted the Budapest Declaration, which prioritizes a set of actions that Member States can start implementing immediately, and proposes steps to strengthen governance, human resources, financing and knowledge for health and the environment.

https://bit.ly/3XLP9w0

## Belize certified malaria-free

WHO certified Belize to be malaria-free. The country has seen cases fall from a peak of about 10 000 cases in 1994 to zero indigenous cases since 2019.

The efforts of community health workers to ensure access to timely diagnosis and treatment were crucial to the country’s success, as were robust surveillance, and effective vector control methods including insecticide-treated mosquito nets and indoor spraying of insecticides. The country’s engagement with regional and subregional initiatives has also driven progress.

The 21 June announcement brought the number of countries and territories certified as malaria-free to 43 worldwide, including 11 countries in the Region of the Americas.

https://bit.ly/448gn2r

## Pandemic impact on hunger

Over 122 million more people are facing hunger in the world due to the COVID-19 pandemic, repeated weather shocks and conflicts, including the war in Ukraine, according to the latest *State of food security and nutrition in the world* report published by five UN agencies.

Published on 12 June, the report reveals that between 691 and 783 million people faced hunger in 2022. The African region remains the worst affected, with 1 in 5 people facing hunger on the continent, more than twice the global average.

https://bit.ly/3pQccJJ

## Boosting investment in primary health care

Three multilateral development banks joined with WHO to launch a health impact investment platform.

Launched on 23 June during the summit for a new global financing pact that was held in Paris, France, the platform is aimed at investing in and strengthening essential, climate and crisis-resilient primary health-care services in low- and middle-income countries.

The platform will make an initial 1.5 billion euros available in concessional loans and grants to countries to expand the reach and scope of their primary health-care services, with a focus on the most vulnerable and underserved populations and communities.

WHO will act as policy coordinator, ensuring alignment of financing decisions with national health priorities and strategies.

https://bit.ly/3D0r2QW

## Protecting children from harmful foods

WHO released new guidance on policies to protect children from the harmful impact of food marketing. Released on 3 June, the guidance is based on reviews of recent evidence regarding the ways in which food marketing affects children’s food-related attitudes, beliefs and behaviours, and includes a systematic review of evidence regarding food marketing policies.

The guidance recommends that countries implement comprehensive, mandatory policies to protect children of all ages from the marketing of foods and non-alcoholic beverages that are high in saturated fatty acids, trans-fatty acids, free sugars and/or salt.

https://bit.ly/447AWw3

## Antimicrobial resistance research

WHO published its first global research agenda for scientists addressing antimicrobial resistance (AMR). Published on 22 June, the agenda was developed based on an evidence review that identified 40 key topics relating to drug-resistant pathogens.

“This first research agenda from WHO will provide the world’s AMR researchers and funders with the most important topics to focus on, and give the world its best chance to combat AMR,” said Dr Silvia Bertagnolio, Unit Head in WHO’s AMR Division.

https://bit.ly/44bosDm

## Digital interoperability

WHO and Health Level Seven (HL7) International, a non-profit digital health standards development organization, signed an agreement to support the adoption of open interoperability standards for digital health systems.

Signed on 3 July, the agreement will allow WHO and HL7 International to strengthen implementation of the WHO *Global strategy on digital health 2020-2025* at country level, and build capacity to support the adoption and appropriate use of interoperability standards in Member States in an equitable manner.

The introduction of interoperability standards is crucial to optimizing use of health data, and facilitating continuity of care at all levels of health systems.

https://bit.ly/3NWKlRm

**Cover photo**

A student responding to the lecturer’s questions during a lesson at the Armenian State Institute of Physical Culture and Sport, Yerevan, Armenia.

**Figure:**
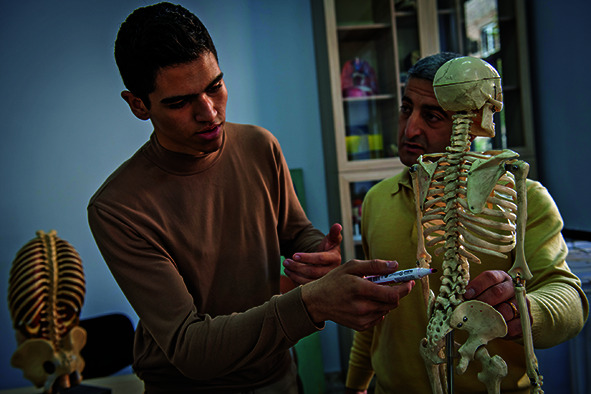
WHO/Nazik Armenakyan

## Gender, water and sanitation

Globally, girls are nearly twice as likely as boys to bear the responsibility for fetching water for households, according to a new report released by the United Nations Children’s Fund and WHO.

Released on 6 July, *Progress on household drinking water, sanitation and hygiene (WASH) 2000-2022: special focus on gender*, provides the first in-depth analysis of gender inequalities in WASH – and sets out their multiple implications for women and girls’ health.

https://bit.ly/43m6XiL

## Aspartame assessments

Assessments of the health impacts of the non-sugar sweetener aspartame were released by the International Agency for Research on Cancer (IARC) and the WHO/FAO Joint Expert Committee on Food Additives (JECFA)on 14 July.

Citing “limited evidence” for carcinogenicity in humans, the IARC classified aspartame as possibly carcinogenic to humans, and the JECFA reaffirmed the acceptability of a daily intake of 40 mg/kg body weight.

https://bit.ly/46QVcDG

**Looking ahead**

17–18 August 2023. First WHO Traditional Medicine Global Summit. Gandhinagar, Gujarat, India. https://bit.ly/3oICahK

29­–31 August 2023. WHO Global Evidence-to-Policy Summit 2023. Virtual event. https://bit.ly/3OcMTuR

5–6 September 2023. Second WHO Symposium on the Future of Digital Health Systems in the European Region. Porto, Portugal. https://bit.ly/3PHBd4o

